# Supplementary material for: Influence of bodyweight on prednisolone pharmacokinetics in dogs
Source: PLoS One. 2025 Jul 8;20(7):e0326586. doi: 10.1371/journal.pone.0326586 (PMC12237026; doi:10.1371/journal.pone.0326586)
Supplement: S1 Table — Optimised MRM parameters for Prednisolone and Prednisolone-D6. In the ‘Product m/z’ column, ‘Q’, ‘R1’ and ‘R2’ indicate the quantifier, and first and second qualifier ions respectively. (DOCX) [file pone.0326586.s002.docx]

**S1:** Optimised MRM parameters for Prednisolone and Prednisolone-D6.

| **Precursor *m/z*** | **Product *m/z*** | **Dwell Time (ms)** | **Collision Energy (V)** | **Q1 Pre-bias (V)** | **Q3 Pre-bias (V)** |
| --- | --- | --- | --- | --- | --- |
| **Prednisolone** | | | | | |
| 361.2 | 147.05 (Q) | 50 | -25 | -25 | -16 |
| 361.2 | 223.1 | 50 | -20 | -24 | -25 |
| 361.2 | 307.25 (R2) | 50 | -25 | -13 | -21 |
| 361.2 | 325.2 (R1) | 50 | -28 | -13 | -23 |
| 361.2 | 343.2 | 50 | -15 | -11 | -24 |
| **Prednisolone-D6** | | | | | |
| 367.2 | 150 (Q) | 50 | -13 | -28 | -15 |
| 367.2 | 226.1 | 50 | -28 | -21 | -27 |
| 367.2 | 312.2 (R2) | 50 | -23 | -12 | -23 |
| 367.2 | 330.2 (R1) | 50 | -25 | -12 | -23 |
| 367.2 | 349.2 | 50 | -11 | -10 | -25 |

In the ‘Product m/z’ column, ‘Q’, ‘R1’ and ‘R2’ indicate the quantifier, and first and second qualifier ions respectively.
