# Supplementary material for: Influence of bodyweight on prednisolone pharmacokinetics in dogs
Source: PLoS One. 2025 Jul 8;20(7):e0326586. doi: 10.1371/journal.pone.0326586 (PMC12237026; doi:10.1371/journal.pone.0326586)
Supplement: S2 Table — (DOCX) [file pone.0326586.s003.docx]

**S2:** Intraday and interday precision and accuracy for the determination of prednisolone concentrations in dog plasma.

|  |  | **Intraday** | | |  | **Interday** | | |
| --- | --- | --- | --- | --- | --- | --- | --- | --- |
| **Pred Conc.**  **(ng·mL^-1^)** |  | **Measured Conc.**  **(mean)** | **Accuracy**  **(%)** | **Precision**  **(% CV)** |  | **Measured Conc.**  **(mean)** | **Accuracy**  **(%)** | **Precision**  **(% CV)** |
| 1 |  | 1.2 | 116.7 | 6.1 |  | 1.1 | 106.8 | 8.3 |
| 3 |  | 3.1 | 102.9 | 6.4 |  | 2.9 | 96.1 | 7.4 |
| 10 |  | 9.2 | 92.0 | 6.4 |  | 9.0 | 89.5 | 11.2 |
| 20 |  | 20.2 | 101.1 | 6.1 |  | 20.5 | 102.7 | 6.5 |
| 50 |  | 45.7 | 91.5 | 4.5 |  | 43.9 | 87.8 | 2.4 |
| 100 |  | 96.3 | 96.3 | 4.0 |  | 95.6 | 95.6 | 5.8 |
| 200 |  | 191.3 | 95.6 | 5.3 |  | 204.0 | 102.0 | 2.5 |
| 250 |  | 244.4 | 97.8 | 3.2 |  | 248.4 | 99.4 | 2.0 |
| 500 |  | 514.1 | 102.8 | 2.2 |  | 500.6 | 100.1 | 0.6 |
