# Supplementary material for: Influence of bodyweight on prednisolone pharmacokinetics in dogs
Source: PLoS One. 2025 Jul 8;20(7):e0326586. doi: 10.1371/journal.pone.0326586 (PMC12237026; doi:10.1371/journal.pone.0326586)
Supplement: Attachment — Submitted filename: 250327_Response_to_reviewers_final.docx [file pone.0326586.s005.docx]

Dear Drs Zabel, Johnson, and Griffenhagen,

Thank you for reviewing our manuscript and your constructive feedback. Please, find below our reply point by point. We are also uploaded the following documents:

- Cover letter (reviewed)
- Response to reviewers
- Marked-up copy of the revised document (Revised Manuscript with Track Changes)
- Unmarked version (Manuscript)

Journal Requirements:

When submitting your revision, we need you to address these additional requirements.

1. Please ensure that your manuscript meets PLOS ONE's style requirements, including those for file naming. The PLOS ONE style templates can be found at <https://journals.plos.org/plosone/s/file?id=wjVg/PLOSOne_formatting_sample_main_body.pdf> and <https://journals.plos.org/plosone/s/file?id=ba62/PLOSOne_formatting_sample_title_authors_affiliations.pdf>

The style has been revised as per requirements.

2. We note that the grant information you provided in the ‘Funding Information’ and ‘Financial Disclosure’ sections do not match.

When you resubmit, please ensure that you provide the correct grant numbers for the awards you received for your study in the ‘Funding Information’ section.

Our study was only supported by one grant from the ACVIM: ACVIM grant - Ref: 775788. This has been added to the cover letter.

3. Thank you for stating the following financial disclosure:
American College of Veterinary Internal Medicine Resident Research Grant

Please state what role the funders took in the study. If the funders had no role, please state: "The funders had no role in study design, data collection and analysis, decision to publish, or preparation of the manuscript."

If this statement is not correct you must amend it as needed.

This statement is correct and was added to the cover letter.

4. When completing the data availability statement of the submission form, you indicated that you will make your data available on acceptance. We strongly recommend all authors decide on a data sharing plan before acceptance, as the process can be lengthy and hold up publication timelines. Please note that, though access restrictions are acceptable now, your entire data will need to be made freely accessible if your manuscript is accepted for publication. This policy applies to all data except where public deposition would breach compliance with the protocol approved by your research ethics board. If you are unable to adhere to our open data policy, please kindly revise your statement to explain your reasoning and we will seek the editor's input on an exemption. Please be assured that, once you have provided your new statement, the assessment of your exemption will not hold up the peer review process.

- We have elected to use github for data sharing. The data has already been uploaded and the link will be made live at the time of publication. It will include:
  - Code of the model used.
  - LCMS data.
- <https://github.com/APWoodward/prednisolone_PK>

 5. [Please include captions for your Supporting Information files at the end of your manuscript, and update any in-text citations to match accordingly. Please see our Supporting Information guidelines for more information: http://journals.plos.org/plosone/s/supporting-information.](https://eur02.safelinks.protection.outlook.com/?url=http%3A%2F%2Ftrack.editorialmanager.com%2FCL0%2Fhttp%3A%252F%252Fjournals.plos.org%252Fplosone%252Fs%252Fsupporting-information.%2F1%2F010f0195879b33bd-27390589-d6a9-4632-955f-ddffb4d9166f-000000%2FSlWkuxDechDe0GneVTX_vnLRf1WnDzujOQ9Wp-yQMDc%3D201&data=05%7C02%7C%7C9767f96c667f4e63dd5308dd60f71d45%7C2e9f06b016694589878910a06934dc61%7C0%7C0%7C638773336945836820%7CUnknown%7CTWFpbGZsb3d8eyJFbXB0eU1hcGkiOnRydWUsIlYiOiIwLjAuMDAwMCIsIlAiOiJXaW4zMiIsIkFOIjoiTWFpbCIsIldUIjoyfQ%3D%3D%7C0%7C%7C%7C&sdata=MVl%2FiG0rPbgxdvCF7k%2B1%2FJ4gZU2wdw%2BOQl1xbnllpgk%3D&reserved=0)

- The supporting information files have been amended accordingly.

6. Please review your reference list to ensure that it is complete and correct. If you have cited papers that have been retracted, please include the rationale for doing so in the manuscript text, or remove these references and replace them with relevant current references. Any changes to the reference list should be mentioned in the rebuttal letter that accompanies your revised manuscript. If you need to cite a retracted article, indicate the article’s retracted status in the References list and also include a citation and full reference for the retraction notice.

- Reference 16 has been updated (from pre-print to published).

Reviewers' comments:

Reviewer's Responses to Questions

**Comments to the Author**

Reviewer #1: This study is relevant to current veterinary practices and seeks to answer a question regarding prednisolone dosing in dogs that has long been a source of debate. The pharmacokinetics were analyzed appropriately and the study design was valid although as the authors point out a longer sampling time would have been useful due to the variability of absorption. The authors comment on variation in body condition score and I would be very curious to see the pharmacokinetic data with respect to body condition score in addition to weight. I would recommend that the authors insert a table or preferably figure with the BCS of the animal as well as the AUC as prepared in Figure 1. Although a firm recommendation on dosing is not possible from this study it is a good start to answer an important physiologic question.

Thank you for the positive feedback to our manuscript and the suggestion of inclusion of the body condition score to our data.

From the physiologic perspective the relevance of the body condition score would be in the relationship between body weight and some notion of ‘body size’, with the hypothesis that the scaling of organ function is directly with body size rather than body weight. This may explain some of the remaining between-subject variation in our analysis, as the bodyweight is an imperfect surrogate for the true predictor ‘body size’.

Although this is reasonable in principle, it would be statistically challenging for various reasons. Particularly important would be the rigor of body condition scoring measurement process; as the measure is subjective and generally vague in the clinical usage, careful prospective planning and justification would be necessary for meaningful results. The high potential for collinearity of the body weight and body condition score suggests that even under good measurement conditions an exaggerated sample size would be needed to tease out the direct effects simultaneously, and this may be difficult to predict in advance, especially if balance in the distribution of body weight and BCS couldn’t be assured.

This is especially true in our current set of dogs, with 19 out of 25 dogs having a BCS of 5 or 6 (S1. File)

We feel that it is doubtful that the data at hand would provide meaningful information about BCS due to these limitations. For this reason, we would respectfully suggest not including such a figure.

Reviewer #2: The authors present an interesting study on the effects of bodyweight based prednisolone dosing in dogs. It is indeed unfortunate that no real conclusions can be generated other than discussing the wide variations in pharmacokinetics between dogs.
From looking at the plasma concentrations in Figure 1, it appears that there are 3 populations of dogs here: The classical early peak followed by a long declining tail, a biphasic group, and a group that never reaches peak concentrations in the sampled interval. Did you separate these out to see if there were any defining factors that could explain this variation - drugs, GI disease, etc.

Thank you for your thoughtful review of our manuscript. Dogs with chronic enteropathy (including protein-losing enteropathy) had a classical absorption. This is consistent with a recent study from Dr Jablonski (DOI: 10.1111/jvim.17277) that reported no difference in PK between dogs with PLE and healthy dogs.

There are some major statistical problems with attempting to explain emergent patterns in the data after-the-fact. This is essentially a form of HARKing. Because the study was not designed with these effects in mind there is a substantial risk that these types of associations have no causal interpretation, and also that any such comparisons lack much power. This suggests that any patterns observed through this sort of reasoning will be unstable and will not generalize. This process also embeds researcher confirmation bias, because the observed effect (the data pattern) and the proposed explanation emerged from the same data. It is reasonable to suggest, based on *a priori* reasoning, what effects might have contributed, but not to try and assess those based on these data. For these reasons, our preference is not to further separate the groups, but this should be kept in mind for future studies.

I have concerns about trying to model PK when there are such obvious differences in bioavailability and absorption patterns between subjects.

This concern presumes that variation in bioavailability and absorption presents some validity threat. I’m not sure why this should be the case. This perspective would generally preclude inference from pharmacokinetic data except under closely controlled experimental conditions, which defeats the whole notion of population PK. Here, we chose to conduct the inference using a noncompartmental approach, which is invariant to the absorption process. Though the incompleteness of observation of some subjects does limit the accuracy of the final analysis, it is not clear why this should preclude doing statistical inference altogether.

It is important to keep in mind that if there is large between or within-subject variation in bioavailability under field conditions, this has a strong influence on the contextual relevance of the systematic effect of bodyweight on exposure (in statistical terms, the ‘effect size’). The opportunity to assess that to some extent is actually a relative advantage of this design compared to working with experimental animals as in most veterinary PK studies, which artificially constrain variation.

In the abstract (line 37) you note that a non-linear regression model 'described the relationship', but you weren't actually able to effectively model the relationship between BW and plasma concentrations. Did you apply a model, or attempt to model, or other? Perhaps just a language 'thing', but it reads to me like you were successful in fully modelling the relationship. Also in the abstract, the coefficients in the mathematical expression are lower case, but the description of them uses upper case.

We agree that this is likely predominantly an issue of terminology. We have attempted to be as clear and transparent as possible about the modelling workflow throughout the manuscript. I’m not sure what is meant by the phrases ‘apply a model’ or ‘attempt to model’. The methodology specifies exactly what models were generated and how they were generated, and their findings are presented in multiple forms in the results.

- The phrase in the abstract ‘described the relationship’ is simply an articulation of what was done. It conveys no claim about how much was learned from doing so or that anything of interest was demonstrated or proved.
- The completed nonlinear regression model which described AUC as a function of bodyweight and dose captured a meaningful amount of the total variability (Bayes R^2: 74.9). The extent to which this is ‘effective’ is entirely subjective, but it is objectively the case that the degree of variation described by the model was large. That the available data did not contain sufficient evidence to decisively support the value of key parameters is a different consideration.
- There is no standard usage of the term ‘fully modelling’. The statistical model includes features that capture systematic effects of the predictors of interest, and remaining unexplained randomness. These are ‘full’ in the sense that they provide a description of the data that is apparently internally valid, and addressed the questions of interest.

Thank you for noticing the discrepancy in the coefficients. We prefer the upper case, so have revised them for consistency throughout.

You spend a fair amount of time discussing BSA in the introduction, but none of your aims involve this term. Did you set out to determine if BSA based dosing was a better method? Or are you just using this as an example of allometric scaling? I also don't see specifically where you modeled both dose in mg/kg and dose in mg/m2?

The common dosage scaling as a ratio of body weight ‘per kilogram’ assumes a simple linear relationship between dose and exposure, *i.e.* *B* = 1. Scaling of dosage by body surface area is a popular alternative in clinical applications, especially in oncology, and assumes *B* = 2/3. It has been recently suggested to use body surface area when dosing prednisolone for large dogs diagnosed with immune-mediated haemolytic anaemia (DOI: 10.1111/jvim.15463) as they apparently developed more fequent adverse effects from prednisolone.

These are two special cases for the general allometric model. Under the hypothesis that larger dogs experience relative overdosing because *B*<1, it would be clinically convenient, and biologically interesting, if *B* = 2/3 were an accurate approximation (the data at hand suggest that it is not). A potential misinterpretation of this system, that is important to address, is that ‘bodyweight’ and ‘body surface area’ dosing represent discrete choices, but actually each are just specific examples of an underlying continuous model, and neither may apply.

Body surface area can be obtained as a simple allometric function of body weight (kg), using the expression $0.101\times{BW}^{2/3}$, as expressed in the manuscript. So, any prednisolone dosage expressed as mg/kg can be expressed equivalently as mg/m^2^, and vis-versa, by simple conversion, if body weight is known. The statistical model describes AUC as a function of dosage ‘mg’ and bodyweight ‘kg’, so the generating predictions regarding dosage in mg/kg and mg/m^2^ was done simply by manipulating the input variables. No separate statistical modelling is required, and would be inappropriate because such models would be implicitly closely related and not separately interpretable.

Line 89 - what did you use as criteria for 'clinically apparent liver disease'?

Thank you for this comment, dogs had either no evidence of synthetic failure or normal bile acid stimulation test (e.g. rule out of significant liver disease, for example for the dog with protein-losing enteropathy). The following has been added to clarify this further:

- Line 89: (no evidence of synthetic failure or normal bile acid stimulation test)

Lines 88 and 101 - One of the criteria for inclusion was that the dogs were amenable to intravenous catheter placement, then you say that you placed an intravenous catheter, but later on you note that some dogs did not get an intravenous catheter (line 116). Which was it?

Thank you for highlighting this discrepancy. We had ethics set out in case not all dogs were amenable to catheter placement, but they actually were (samples collected are listed in S. file 1). We have removed line 116

Did you see if there were differences in PK for those that received the pill in for vs those that didn't (line 106).

Most dogs were given the pill in a small meat ball or with a piece of chicken. No pattern was noted and although it is reasonable to pose this as a potential contributor, we would not be able to assess this further with the current dataset.

Line 114 - How many of the patients got all 10 samples collected. You note that many of them did not for various reasons, but not how many actually completed the study.

This has been clarified in the manuscript (line 254):

“A total of 16 dogs had 8 or more samples including two dogs with 10 samples S1 File 1. For the majority of dogs, less than ten samples were collected when the sampling catheter became non-patent and only two additional direct venepunctures were obtained as approved by our ethics committee. In a few instances, dog owner schedule limitations, or other practical imitations on the day of the study prevented to obtain all samples.”

The data are fully expressed for the reader in S1 file as well as figure 1, along with the goodness-of-fit of the accompanying model, which provides the reader with the ability to see exactly what data were being used and how the number and distribution of observations for each subject affects the results.

In your quantification section, I would suggest referring to your methods as 'liquid-chromatography tandem mass spectrometry (LC-MS/MS)' as in the abstract for consistency (and technical correctness).

Thank you for this comment, we have adjusted the manuscript for consistency as suggested.

I am no mathematician, so I could be wrong on this, but you seem to have separated the terms on the right side of Equation 2 using both log base 10 and natural log together. Is this technically correct?

Thank you for noticing this mistake. You are right, there is actually no usage of the base 10 logarithm anywhere in the model. In equation 3 there was a typographic error and the character *e* was missed; this has been corrected in the updated submission.

Line 238 - you note that only 25 of the 26 dogs contributed to data analysis, but there 26 curves in Figure 1, and in fact the legend for Figure 1 indicates that you are presenting data from 25 dogs.

Figure 1 includes only 25 curves. The following sentence has been added in the manuscript to clarify (Line 287):

“Dog 4 was excluded from data analysis due to delayed absorption and therefore not included in this figure.”
